# Ex vivo generation of platelet products from human iPS cells

**DOI:** 10.1186/s41232-020-00139-2

**Published:** 2020-12-01

**Authors:** Sou Nakamura, Naoshi Sugimoto, Koji Eto

**Affiliations:** 1 Department of Clinical Application, Center for iPS Cell Research and Application, Kyoto University, 53 Kawahara-cho, Shogoin, Sakyo-ku, Kyoto, 606-8507 Japan; 2 Department of Regenerative Medicine, Chiba University Graduate School of Medicine, 1-8-1 Inohana, Chuo-ku, Chiba-shi, Chiba, 260-8677 Japan

**Keywords:** Platelet, Megakaryocyte, iPS cell, Turbulence, Bioreactor

## Abstract

Platelet products are used in treatments for thrombocytopenia caused by hematopoietic diseases, chemotherapy, massive hemorrhages, extracorporeal circulation, and others. Their manufacturing depends on volunteers who donate blood. However, it is becoming increasingly necessary to reinforce this blood donation system with other blood sources due to the increase in demand and shortage of supply accompanying aging societies. In addition, blood-borne infections and alloimmune platelet transfusion refractoriness are not completely resolved. Since human induced pluripotent stem cell (iPSC)-platelet products can be supplied independently from the donor, it is expected to complement current platelet products. One big hurdle with iPSC-based systems is the production of 10 units, which is equivalent to 200 billion platelets. To overcome this issue, we established immortalized megakaryocyte cell lines (imMKCLs) by introducing three transgenes, c-MYC, BMI1, and BCL-XL, sequentially into hematopoietic and megakaryocytic progenitor stage cells derived from iPSCs. The three transgenes are regulated in a Tet-ON manner, enabling the addition and depletion of doxycycline to expand and maturate the imMKCLs, respectively. In addition, we succeeded in discovering drug combinations that enable feeder-free culture conditions in the imMKCL cultivation. Furthermore, we discovered the importance of turbulence in thrombopoiesis through live bone marrow imaging and developed a bioreactor based on the concept of turbulent flow. Eventually, through the identification of two key fluid physic parameters, turbulent energy and shear stress, we succeeded in scaling up the bioreactor to qualitatively and quantitatively achieve clinically applicable levels. Interestingly, three soluble factors released from imMKCLs in the turbulent flow condition, macrophage migration inhibitory factor (MIF), insulin growth factor binding protein 2 (IGFBP2), and nardilysin (NRDC), enhanced platelet production. Based on these developments, we initiated the first-in-human clinical trial of iPSC-derived platelets to a patient with alloimmune platelet transfusion refractoriness (allo-PTR) using an autologous product. In this review, we detail current research in this field and our study about the ex vivo production of iPSC-derived platelets.

## Background

Platelets are released from megakaryocytes (MKs) in the bone marrow and circulate as anucleate blood cells with a diameter of about 2–4 μm. Platelets are essential for hemostasis, and their reduction or dysfunction causes various bleeding complications. During normal platelet production, the cytoplasm of mature MKs exhibits a stretched morphology. This rapid morphological change is called proplatelet formation, and the extended tip of which is sheared by the bloodstream, releasing over a thousand platelets [1–4]. In emergencies, when a large amount of platelets is required urgently, MKs rupture depends on interleukin-1 alpha (IL-1α) regulation and releases a large amount of platelets at once [5]. Recently, MKs were also reported in lung and spleen tissues, where platelets are released by the blood flow in the lung and spleen vasculatures [6].

In the clinic, the treatment of diseases and conditions that cause cytopenias heavily depends on blood donation systems. However, blood donation systems are becoming insufficient due to increasing demand and decreasing numbers of blood donors in aging societies. Contamination by blood-borne pathogens is also not completely resolved due to vulnerabilities in testing due to the so-called window period of infections and emerging infections. Furthermore, with regards to platelet products, the shelf life is only 4 days because platelet function falters thereafter and bacterial growth is a risk due to storage at room temperature, thus causing added challenges to maintain the balance of the supply and demand. Moreover, alloantibodies against human leukocyte antigen class I (HLA-I) and to a lesser extent to human platelet antigen (HPA) cause alloimmune platelet transfusion refractoriness (allo-PTR) in 5–15% of patients receiving platelet transfusion. These patients with allo-PTR require HLA-I/HPA-matched platelet transfusions, presumed to be more than 20,000 bags of platelets per year in Japan, which increases the risk of supply shortages due to limited donors or even complete unavailability in the case of rare types and emergencies [7]. For these reasons, the development of blood transfusion products independent of blood donation has been receiving attention. One example is platelets generate ex vivo from induced pluripotent stem cells (iPSCs) [8]. From the point of view of regenerative medicine, the non-tumorigenicity of iPSC-derived platelets can be easily ensured, because platelets are anucleate cells and irradiation before transfusion will eliminate the tumorigenic potential of any contaminating nucleate cells.

In addition, a system that stably supplies HLA-compatible platelets can be constructed by using the patients themselves or iPSCs having various HLA. Furthermore, iPSCs established from a specific patient can be a powerful tool for disease elucidation and drug development by reproducing the disease in vitro. In this review, we summarize the development ex vivo iPSC-derived platelets with remarks on expandable MKs lines, bioreactor systems, and newly discovered reagents.

## Megakaryocyte differentiation from iPSCs

For platelet transfusions in Japan, 10 units (about 200 billion platelets) are transfused once per transfusion unless the hematopoietic function is restored. In other words, a large-scale culture is essential for the production of platelet products using human iPSCs. Because iPSCs can infinitely proliferate in vitro and differentiate to MKs and eventually platelets, it was thought that a large amount of platelets could be obtained by amplifying a large number of iPSCs. Takayama et al. successfully produced platelets from human ES cell and individual human iPSC clones [9, 10]. These human iPSC-derived platelets showed clot formation in vivo [10]. Feng et al. generated platelets from human iPSCs in the no serum and animal feeders free. They succeeded in large-scale culture of 1.26 × 10^8^ iPSCs to generate 2 × 10^9^ megakaryocyte progenitor cells [11]. However, no study has expanded iPSCs and then differentiated them platelets to sufficient levels for clinical trials. This is because MK differentiation cultures are long, complex, costly processes. As a solution, we established immortalized MK cell lines (imMKCLs) as direct progenitors of platelets.

## Establishment of immortalized megakaryocyte progenitor cell lines from iPSCs

Recently, in adult hematopoiesis, MK progenitor cells, which are the precursors of MKs, were suggested to differentiate directly from hematopoietic stem cells or pluripotent blood progenitor cells [12–14]. This finding indicates the existence of MK lineage-committed early progenitors that are capable of self-renewing.

Based on the construction of a differentiation culture system for in vitro human embryonic stem cells (ESCs) and iPSCs, we clarified that changes in the expression of the cell cycle regulator c-MYC at the MK differentiation stage regulate the growth and maturation processes of MKs [10]. In other words, we proposed that c-MYC is required during the proliferative stage of MK progenitor cells, but, at the same time, its expression must be suppressed during the MK maturation stage. Based on this knowledge, we established imMKCLs by the sequential introduction of c-MYC and BMI1 genes followed by the BCL-XL gene into human iPSC-derived hematopoietic progenitor cells [15]. These transgenes are under the control of the Tet-on system, and doxycycline (DOX) switches imMKCLs from the growth stage (DOX-on) to the maturity stage (DOX-off). Since the overexpression of c-MYC induces cellular senescence and apoptosis [16], the polycomb complex component BMI1 and the anti-apoptotic factor BCL-XL are co-expressed to regulate the cellular senescence-inducing genes ARF and INK4A and caspase, respectively [17, 18].

**Fig. 1:**
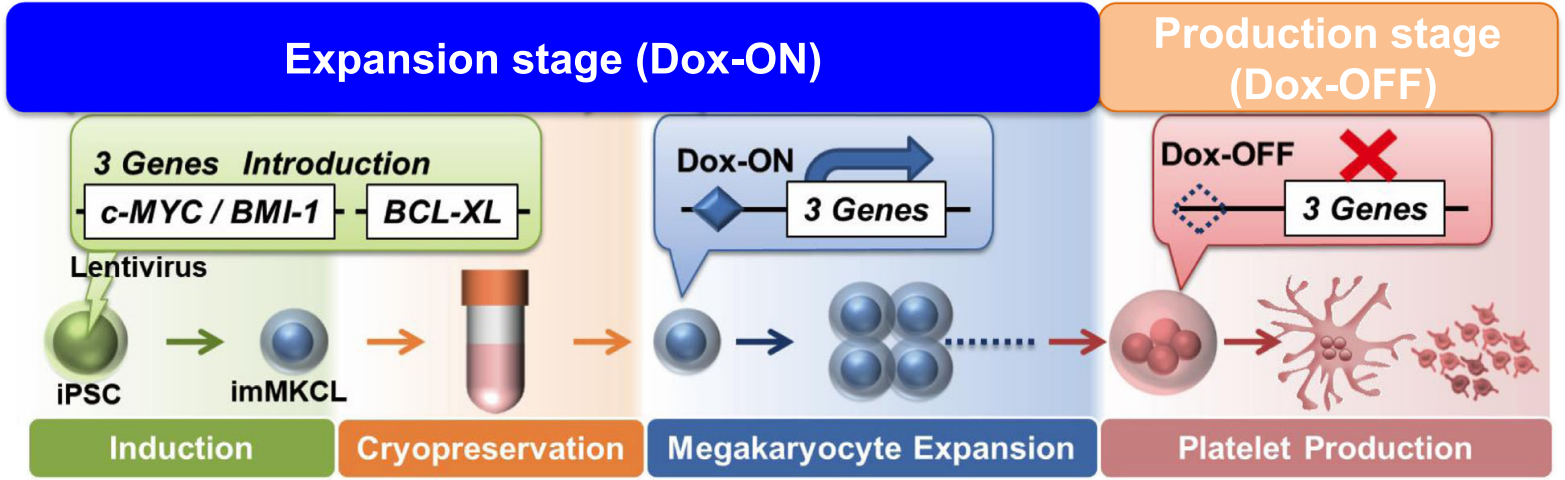
The expansion and production stages of imMKCLs. imMKCLs are established from iPSC-derived hematopoietic progenitor cells by the lentiviral gene transfection of c-MYC, BMI1, and BCLXL. By removing doxycycline (Dox-OFF), imMKCLs can switch from self-renewal to maturation with platelet production. These cells are cryopreserved as a master cell stock, and clinical grade platelets can be produced by performing the manufacturing process after thawing according to good manufacturing practice (GMP) guidelines [15, 25]

When the expression of these transgenes is turned off, the expression levels of the MK maturation factors GATA1, NFE2, FOG1, and β1-tubulin increase [4, 19–21]. Accordingly, proplatelets, a precursor of platelet production, are observed, and vWF receptor CD42b (GPIbα)-positive platelets are released (Fig. 1). imMKCLs are suitable as master cells, because they can be stored frozen and then thawed and expanded for platelet production as needed. Infectious agent-free MK master cells can manufacture platelet products that comply with good manufacturing practices (GMP) by culturing in aseptic culture conditions. One limitation of this approach is that the imMKCLs are not always stably established, which may be due to clonal variations in iPSC, difficulty in introducing optimal level of transgenes, but still unclear yet.

Moreau et al. proposed another approach to obtain expandable MK lines [22]. Here large-scale MKs are generated using a forward programming strategy (fopMKs) that depends on the exogenous expression of three transcription factors, GATA1, FLI1, and TAL1. These factors are introduced into human iPSCs and differentiated into MKs using the embryoid body (EB) method. FopMKs proliferate and differentiate in culture for several months, and TPO in combination with interleukin-1 beta (IL-1β) promotes their maturation and release of platelets. Furthermore, Ono et al. and Pulecio et al. directly converted mouse and human fibroblasts into MK progenitors by genetically introducing three factors, NFE2, MAFG, and MAFK, or six factors, GATA1, GATA2, LMO2, RUNX1,TAL1, and c-MYC [23, 24]. A major problem in the production of platelet products using these cells, however, is the development of large-scale culture methods. In the next section, we introduce our study on this problem.

## Construction of feeder-free imMKCL culture systems

Although expandable, imMKCLs are not efficient at generating platelet-like particles. Consider, one MK in the bone marrow is estimated to produce 800–2000 platelets, whereas imMKCLs generate 3–10 platelets per cell. In addition, the imMKCL culture used feeder cells. For a large-scale culture based on a shaking culture, it is essential to construct a feeder-free culture system. In the expansion stage, it was found that imMKCLs can be cultured in a 100-mL flask and 1–20-L WAVE bag system by mild rocking movement without feeder cells. However, in mature stage, it became apparent that the quality and number of the generated platelets was remarkably low using the WAVE bag system compared to feeder cell cultures. To overcome this issue, we screened candidate drugs and found that an AhR antagonist, StemReginin1 (SR-1), plus a ROCK inhibitor, Y-27632, most efficiently promoted platelet generation under feeder-free conditions [25]. Interestingly, the rotational shaking of a flask significantly increased the number and function of platelets compared with static cultures (culture dish) in this feeder-free method. Therefore, we concluded that physical fluid factors activated by the shaking in the liquid culture condition promote platelet generation.

## Analysis of dynamic blood flow during platelet release in mouse bone marrow

It was previously proposed that blood flow-dependent shear stress is important for platelet biogenesis from mouse MKs in BM [1]. Based on this concept, various types of bioreactors have been designed. For example, Thon et al. generated a microfluidic chip bioreactor that mimics bone marrow, including vascular endothelial cells, extracellular matrix and flow with shear stress. The chip is based on the importance of shear stress in bone marrow environment for proplatelet formation and platelet release [26]. Blin et al. placed VWF-coated micropillars in the microfluidic device to make anchored megakaryocytes susceptible to shear stress and releases platelets [27]. To recreate the bone marrow niche environment of MK, Balduini et al. utilized a silk-based vascular tube containing cytokines, extracellular matrix components, and endothelium-derived proteins [28]. Nakagawa et al. found that different angles correlate with different platelet outputs, suggesting that flow angle may be an important parameter [29]. Avanzi et al. used nanofiber membrane through the pore of which megakaryocytes extend proplatelets for their bioreactors[30]. But the platelet generation efficiency of all remains far short from that of in vivo platelet generation. To investigate the physical factors regulating platelet biosynthesis, we observed dynamic blood flow during platelet release in vivo using two-photon microscopy [5] and particle image velocimetry (PIV). We found that platelets were released from proplatelets at the site where “turbulence” occurred, suggesting turbulence in addition to shear stress are crucial in vivo fluid physical factors for platelet generation.

## Scale-up of culture system by controlling turbulent energy and shear stress

Based on the revised concept incorporating turbulence, we succeeded in the highly efficient generation of iPSC-derived platelets by using VerMES, a newly developed vertical reciprocal motion liquid culture bioreactor capable of generating turbulence. The produced platelets had a quality equivalent to platelets in vivo, as exemplified by the low expression of phosphatidylserine on the extracellular membrane. We performed simulations of the physical parameters to assess their correlation with the platelet generation in VerMES under various motion speed conditions in 0.3 L and 2.4 L scale VerMES. We found that the relationship between the level of turbulent energy and shear stress and the yield of platelets were consistent in the two different scales, indicating the existence of optimal values for the physical parameters that were independent of VerMES volume [25]. By optimizing shear stress and turbulent energy, we succeeded in producing 100 billion healthy platelets in an 8-L VerMES [25]. Morphological observations revealed that the iPSC-derived platelets displayed ultrastructures similar to human platelets, and functional analysis in vitro revealed that granule release, aggregation, and clot retraction were comparable. The post-transfusion kinetics and bleeding time in mouse and rabbit thrombocytopenia models [31] between iPSC-derived platelets and primary platelets were also comparable [25]. Based on these findings, we have started the first-in-human clinical trial of iPSC-derived platelets to a patient with allo-PTR using an autologous product (https://jrct.niph.go.jp/en-latest-detail/jRCTa050190117).

## Mechanism of platelet production in turbulence

**Fig. 2:**
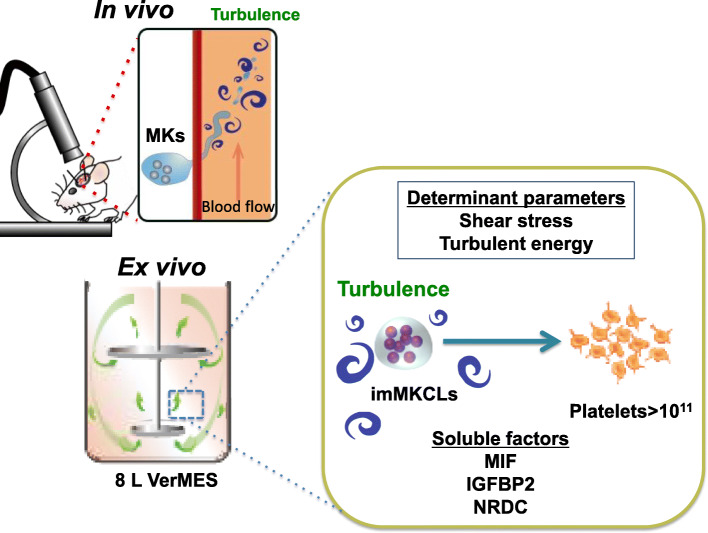
Turbulence promotes platelet generation to allow for the production of 100 billion platelets ex vivo. From in vivo imaging, platelet release was observed at the sites of turbulence in the blood flow. By applying turbulence ex vivo, we succeeded in obtaining 100 million functional platelets from a single imMKCL clone in an 8-L VerMES. Two physical parameters, turbulent energy and shear stress, are involved in the bioreactor scale-up, and turbulence stimulated imMKCL to releases the soluble factors IGFBP2, MIF, and NRDC, which activated the platelet production[25]

To analyze the mechanism of platelet production in the optimal turbulent environment, a microarray analysis of imMKCLs in static and VerMES cultures was performed. However, there was little difference in the gene expression profiles. Next, assuming that soluble factors associated with platelet generation are released from imMKCLs during VerMES culture without changes in gene expressions, the culture supernatant obtained from VerMES culture was added to flask culture, in which turbulence and shear stress are generated but to a lesser level than in VerMES culture, or on a microchip, in which only shear stress exists. The supernatant addition increased the number of platelets. Interestingly, the addition to static culture did not affect platelet production, suggesting that soluble factors in the culture supernatant and shear stress cooperate to contribute to the generation of platelets. According to a proteome analysis of the culture supernatant obtained by VerMES culture, the secreted amounts of insulin growth factor binding protein 2 (IGFBP2) [32], macrophage migration inhibitory factor (MIF) [33], chemokine(C-C motif) ligand 5 (CCL5 or RANTES) [34, 35], thrombospondin-1 (TSP-1) [36], plasminogen activator inhibitor-1 (PAI-1) [37], and nardilysin (NRDC) [38] were all increased compared with the static condition. To investigate the function of these six factors, a microfluidic chip was used to assess platelet generation. This assay revealed that the platelet generation was enhanced with the addition of recombinant proteins of the six factors, but not if any of NRDC, MIF, and IGFBP2 were excluded, suggesting the significance of these three. Replacing NRDC with a mutant defective of zinc-metalloendopeptidase activity also compromised the enhanced production. Since proplatelet formation was significantly reduced in MIF- and IGFBP2-free cultures, we evaluated the extracellular matrix secretion ability of imMKCLs, finding that the defective proplatelet formation was caused by a decreased secretion of vWF and VCAM1 from imMKCLs. On the other hand, immunostaining and immunoprecipitation mass spectrometry analysis revealed that NRDC translocates from the cell nucleus to the plasma membrane during imMKCL maturation and interacts directly with α4A tubulin and β1 tubulin, which are proteins that constitute the microtubules found in proplatelets. Therefore, we suggested that NRDC released in a turbulent environment facilitates shear stress-induced platelet shedding through its zinc-metalloendopeptidase activity [25] (Fig. 2).

## Conclusion

In this review, we provide an overview of the current status of the development of iPSC-derived platelets. The result of this progress has made it possible to generate platelets from human iPSCs that are both clinically applicable in quality and quantity and thus reached a clinical trial. In the future, we plan to scale up the culture tank and improve the culture conditions to enhance the platelet generation performance and further aim for technological innovation to realize industrial production. Ultimately, the aim is to see iPSC-derived platelets provide a transfusion option to all people in a timely manner.

## Data Availability

Not applicable.

## Abbreviations

MKs: Megakaryocytes
PTR: Platelet transfusion refractoriness
HLA: Human leukocyte antigen
HPA: Human platelet antigen
iPSCs: Induced pluripotent stem cells
imMKCL: Immortalized MK progenitor cell line
DOX: Doxycycline
GMP: Good Manufacturing Practice
IGFBP2: Insulin growth factor binding protein 2
MIF: Macrophage migration inhibitory factor
CCL5: Chemokine (C-C motif) ligand 5
PAI-1: Plasminogen activator inhibitor-1
NRDC: Nardilysin
